# Genetic and pharmacological inhibition of CDK9 drives neutrophil apoptosis to resolve inflammation in zebrafish *in vivo*

**DOI:** 10.1038/srep36980

**Published:** 2016-11-11

**Authors:** Laura J. Hoodless, Christopher D. Lucas, Rodger Duffin, Martin A. Denvir, Christopher Haslett, Carl S. Tucker, Adriano G. Rossi

**Affiliations:** 1 MRC Centre for Inflammation Research, The Queen’s Medical Research Institute, The University of Edinburgh, Edinburgh, EH16 4TJ, United Kingdom; 2 BHF Centre for Cardiovascular Science, The Queen’s Medical Research Institute, The University of Edinburgh, Edinburgh, EH16 4TJ, United Kingdom

## Abstract

Neutrophilic inflammation is tightly regulated and subsequently resolves to limit tissue damage and promote repair. When the timely resolution of inflammation is dysregulated, tissue damage and disease results. One key control mechanism is neutrophil apoptosis, followed by apoptotic cell clearance by phagocytes such as macrophages. Cyclin-dependent kinase (CDK) inhibitor drugs induce neutrophil apoptosis *in vitro* and promote resolution of inflammation in rodent models. Here we present the first *in vivo* evidence, using pharmacological and genetic approaches, that CDK9 is involved in the resolution of neutrophil-dependent inflammation. Using live cell imaging in zebrafish with labelled neutrophils and macrophages, we show that pharmacological inhibition, morpholino-mediated knockdown and CRISPR/cas9-mediated knockout of CDK9 enhances inflammation resolution by reducing neutrophil numbers via induction of apoptosis after tailfin injury. Importantly, knockdown of the negative regulator La-related protein 7 (LaRP7) increased neutrophilic inflammation. Our data show that CDK9 is a possible target for controlling resolution of inflammation.

Neutrophils are rapidly recruited to sites of inflammation where they perform key cellular functions including the release of inflammatory mediators, phagocytosis of invading organisms, degranulation and even the release of DNA strands to “trap” pathogens (NETosis)^1^. However, these functions must be carefully controlled in order to prevent tissue damage elicited by the neutrophils themselves^2^. Thus, when neutrophilic inflammation is dysregulated, these cells contribute to the damage that occurs in many diseases, including lung diseases^1^,^3^, atherosclerosis^4^, Alzheimer’s disease^5^ and cancer^6^. Manipulation of neutrophilic inflammation is therefore considered to be an important strategy for treatment of such diseases.

While the *in vivo* lifespan of neutrophils is unclear, recent evidence indicates that they have a half-life of as much as 13–19 hours (h) in blood^7^. Neutrophils during an inflammatory response have an extended lifespan compared to neutrophils in the blood in homeostasis^8^. It is important that neutrophils are cleared from the site of inflammation after exerting their pro-inflammatory effects. Apoptosis is regarded as a non-inflammatory, immune-quiescent process in which the cells become functionally down-regulated, and toxic intracellular contents are contained within the plasma membrane then taken up by surrounding phagocytes (especially macrophages)^1^. Uptake of apoptotic cells causes macrophages to change phenotype to release anti-inflammatory and pro-resolution rather than pro-inflammatory mediators, promoting resolution of inflammation and triggering tissue repair mechanisms^2^,^9^. Defective apoptosis and clearance is associated with many inflammatory conditions, including rheumatoid arthritis^10^ and cystic fibrosis^11^. Targeting neutrophil apoptosis is therefore a feasible therapeutic strategy for these conditions.

Previous work from our group has shown that neutrophil apoptosis is driven *in vitro* by pharmacological cyclin-dependent kinase (CDK) inhibitor compounds, such as *R-*roscovitine^12^ and more recently, AT7519^13^. This is thought to be due to inhibition of CDK9 and/or CDK7, which are integral to the formation of the positive transcription elongation factor b (P-TEFb) complex^12^,^14^. This complex is an key regulator in transcription rather than the cell cycle; in particular, it is involved in transcription of Mcl-1, an important neutrophil survival protein^15^,^16^. In addition, P-TEFb is likely involved in the transcription of pro-inflammatory mediators such as TNF^17^. Importantly, several endogenous inhibitors of the P-TEFb complex exist. One example is the La-related protein 7 (LaRP7), a component of the P-TEFb-inhibiting small nuclear ribonucleic protein (snRNP) complex^18^.

CDK inhibitor drugs also enhance resolution of neutrophilic inflammation in mouse models *in vivo*^12^,^13^. Nevertheless, it is currently unknown whether CDK9 (and/or CDK7) inhibition *in vivo* is the target responsible for the neutrophil apoptosis-driving effect of these inhibitors, as mouse knockouts of CDK9 and its associated proteins are embryonically lethal^19^. It is also unclear whether these apoptosis-enhancing effects are relevant to enhancement of resolution.

In the zebrafish (*Danio rerio*), many mechanisms of innate immune function are conserved. It is also a model organism which is amenable to rapid pharmacological and genetic manipulation and is particularly suitable for live *in vivo* imaging of cell behaviour^20^. We and others have previously shown that CDK inhibitor compounds can enhance inflammation resolution in zebrafish after tissue injury^21^,^22^. However, it is not known whether these pro-resolution effects are dependent upon CDK9 inhibition *in vivo*.

Here we show that CDK inhibitors (AT7519, flavopiridol) that target CDK9, and also genetic methods of targeting CDK9 (morpholinos and CRISPR/cas9) enhance the resolution of neutrophilic inflammation in a model of zebrafish tailfin injury by inducing apoptosis. Importantly, we show that knocking down La-related protein 7 (LaRP7, an endogenous negative regulator of the P-TEFb complex) has the opposite effect to knocking down CDK9, and promoted increased neutrophilic inflammation at the site of wounding. In turn, AT7519 treatment could counteract the effect of LaRP7 knockdown. Genetic but not pharmacological targeting of CDK9 delayed macrophage recruitment to the site of wounding. This study represents the first clear demonstration of the pro-resolution role of CDK9 inhibition in neutrophils in an *in vivo* model of inflammation and injury.

## Results

### Neutrophils and macrophages respond to transection of the median tailfin

**Figure 1:**
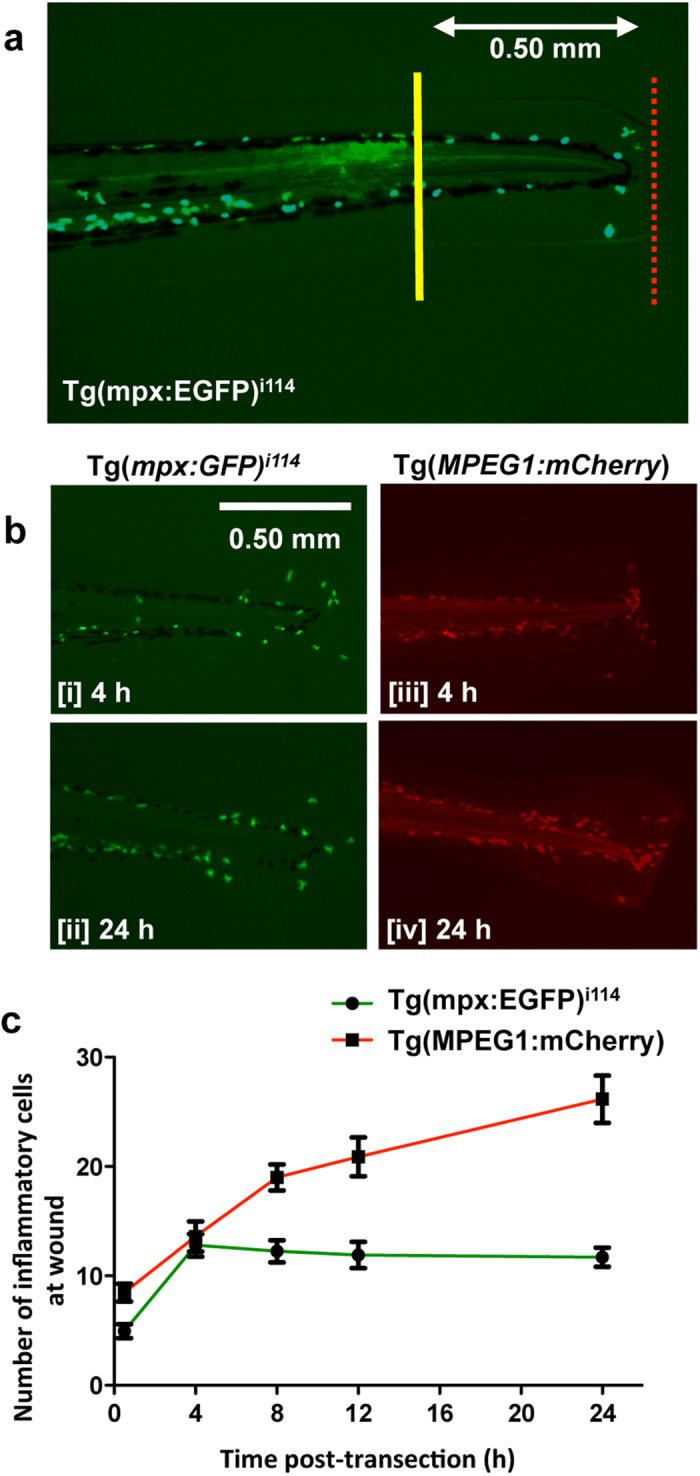
Injury of the zebrafish tailfin results in recruitment of neutrophils and macrophages to the injury site. (**a**) The tailfin of 3 dpf Tg(mpx:EGFP)^i114^ and Tg(MPEG1:mCherry) embryos were transected (line of transection shown in red) and a region (0.5 mm length from the tip of the body of the fish) was selected in which to count recruited cells. (**b**) Temporal recruitment of neutrophils (Tg[mpx:EGFP]^i114^ [i, ii]) and macrophages (Tg[MPEG1:mCherry] [iii, iv]) post-injury was determined. (**c**) The numbers of inflammatory cells in each tailfin region were quantified. All time points after 0 h were significantly (p ≤ 0.05) different to the cell numbers at 0 h. All images at 80x magnification. ≥40 fish per group, from 3 independent experiments. Data expressed as ±S.E.M.

To determine the neutrophil and macrophage response to median tailfin transection, we performed serial imaging of 3 dpf Tg(mpx:EGFP)^i114^ and Tg(MPEG1:mCherry) zebrafish. The line of transection (red dotted) and the area chosen for quantification (cells to the right of the yellow line, drawn at 0.5 mm length from the tip of the body) are depicted (Fig. 1a). Example images and a time-course analysis are shown (Fig. 1b,c). Neutrophil numbers increase after the initial transection, peaking at around 4 h after the initial transection (13 ± 2 cells), before starting to decline at 48 h (data not shown). Macrophage numbers in the Tg(MPEG1:mCherry) line peak at 24 hpi (22 ± 2 cells). In general, macrophages outnumbered neutrophils after injury at the time points studied.

### Pharmacological CDK inhibition reduces established post-wounding neutrophilic inflammation and enhances inflammation resolution

**Figure 2:**
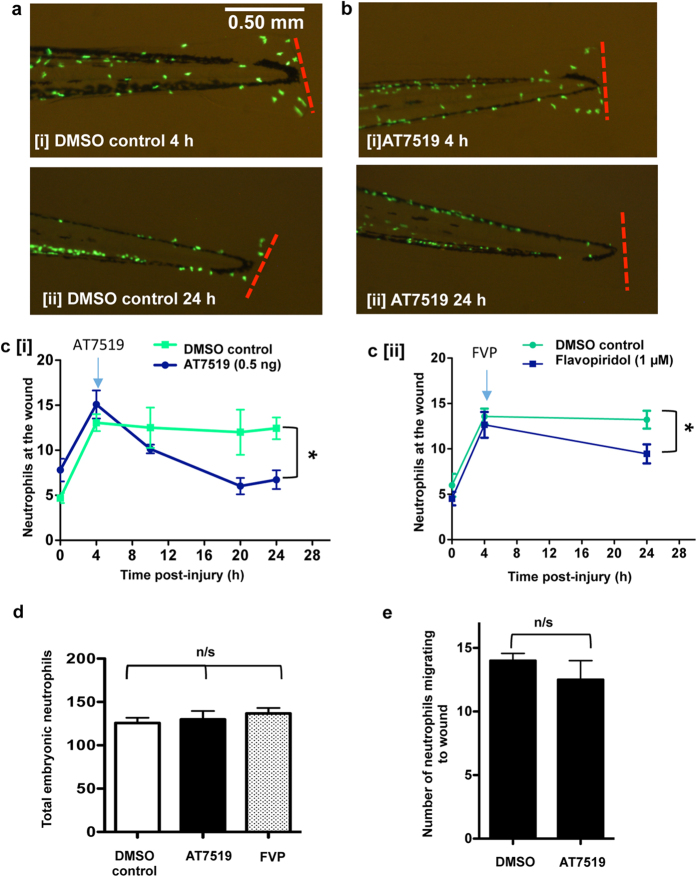
Treatment with AT7519 or flavopiridol accelerates resolution of neutrophilic inflammation. Tg(mpx:EGFP)^i114^ zebrafish embryos underwent tailfin transection at 3 dpf and were serially imaged at various time points post-injury. (**a**) Embryos were micro-injected with DMSO or (**b**) AT7519 at 4 hpi with representative images (80x magnification) from 4 h [i] and 24 h [ii] shown. (**c** [i]) Serial neutrophil numbers at each tailfin were quantified with the blue arrow indicating the 4 hpi time of drug administration. ≥40 fish from 4 independent experiments. Data expressed as ±SEM. (**c** [ii]) In separate experiments, Tg(mpx:EGFP)^i114^ zebrafish embryos were also treated with flavopiridol (FVP) at 4 hpi and the neutrophil numbers at the tailfin quantified. ≥31 fish from 3 independent experiments. (**d**) The total neutrophils in the entire embryo at 24 hpi and DMSO/AT7519/FVP treatment were imaged and quantified. ≥10 fish from 3 independent experiments. (**e**) Time lapse movies of embryos (3 per group) were analysed and the number of cells which migrate to the wound for 15 hpi and DMSO/AT7519 treatment was counted. *p < 0.05 at 24 hpi, data analysed by two-way ANOVA then post-test Newman-Keuls or unpaired t-test. n/s: not significant.

Next, this model was used in conjunction with treatment with CDK inhibitor compounds known to have activity against CDK9 (AT7519 and flavopiridol)^23^,^24^. CDK inhibition with either AT7519 or flavopiridol resulted in a significant reduction in neutrophils at the wound site at 24 hpi (Fig. 2a–c).

To determine if the effects of CDK inhibition were specific to wound site neutrophils, or affected global neutrophil numbers, whole-fish total neutrophil numbers were also quantified. This revealed no significant difference between AT7519-, flavopiridol- or DMSO-treated groups (Fig. 2d). In order to assess the effect of CDK9 inhibition on cell recruitment, we performed time-lapse imaging of embryos from 4 hpi (directly after treatment with AT7519 or DMSO) for a 15 h time period (Fig. 2e). The movies were analysed by tracking how many cells passed into the wound area (as determined in Fig. 1a). The cells were only counted once, therefore only migration is quantified (this discounts any effects on the numbers from apoptosis or reverse migration of neutrophils).

### CDK9 inhibition increases neutrophil apoptosis at the tailfin following wounding

**Figure 3:**
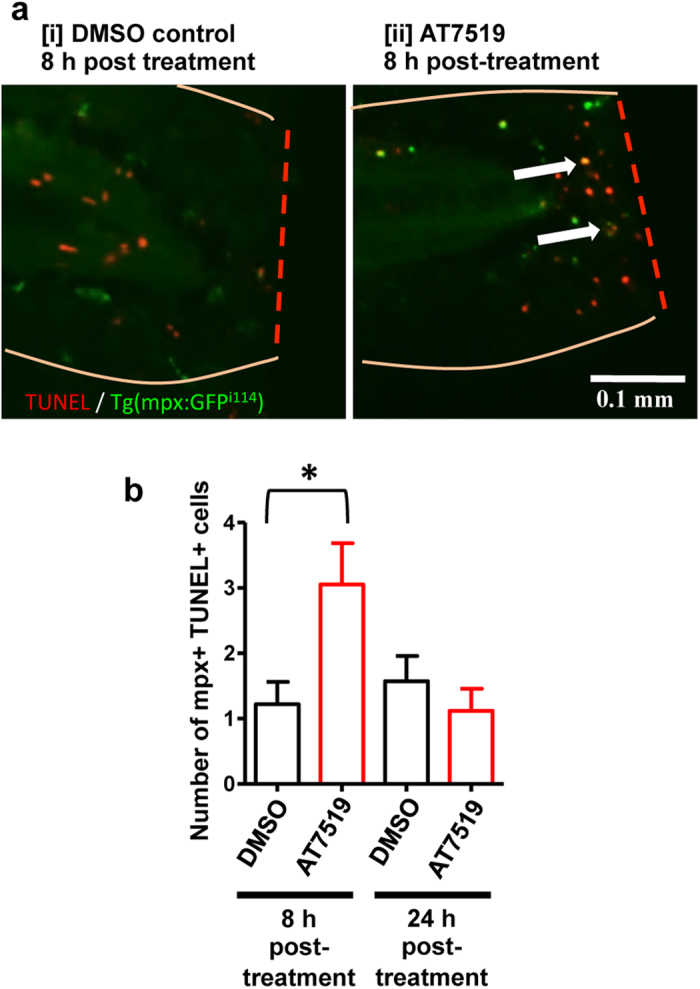
CDK inhibition induces neutrophil apoptosis at the site of tailfin injury *in vivo.* (**a**) TSA/TUNEL staining was carried out on Tg(mpx:EGFP)^i114^ zebrafish following tailfin transection and either DMSO [i] or AT7519 [ii] treatment, labelling neutrophils in green, and apoptotic cells in red, with representative images shown. (**b**) Apoptotic neutrophils (double positive red and green) are shown by the arrows. The absolute number of double positive cells were quantified at 12 hpi. ≥16 embryos per group in 3 independent experiments. *p < 0.05 12 h DMSO vs 12 h AT7519, unpaired t test. n/s: not significant. Data expressed as ±S.E.M.

TSA and TUNEL staining was performed to visualise both neutrophils and apoptotic cells in DMSO control [i] or AT7519 [ii] treated groups, with example images shown (Fig. 3a). At 12 hpi, there were significantly more apoptotic neutrophils in the AT7519-treated group compared to the DMSO-treated control group (Fig. 3b).

### Morpholino-mediated CDK9 knockdown and CDK9 knockout using CRISPR/cas9 enhances resolution of inflammation

**Figure 4:**
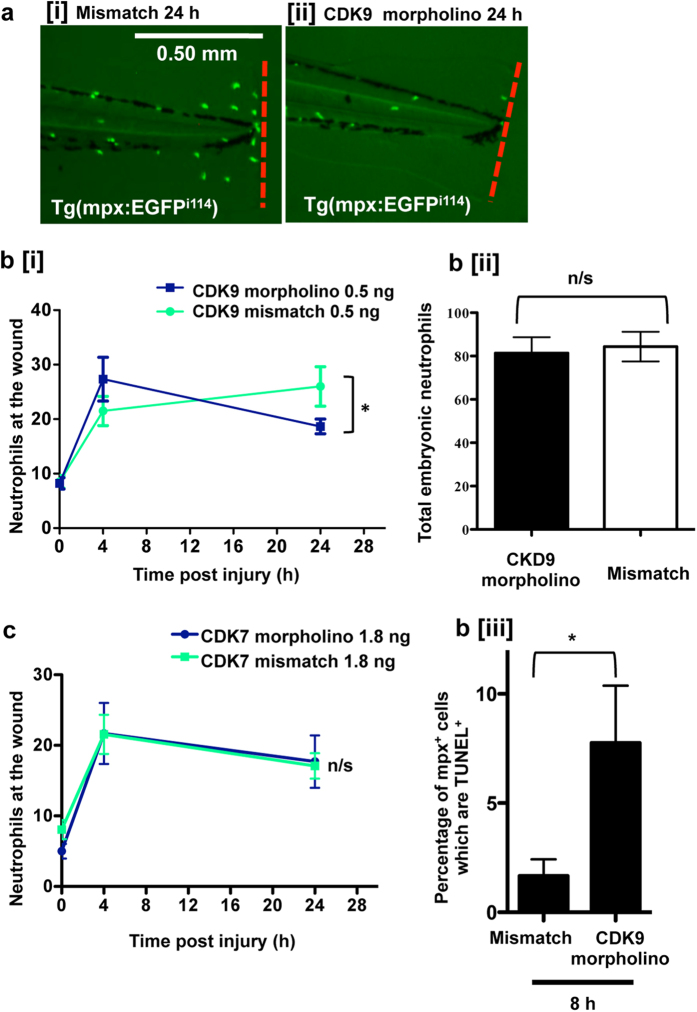
CDK9 knockdown, but not CDK7 knockdown, reduces neutrophilic inflammation. Tg(mpx:EGFP)^i114^ zebrafish eggs were injected with a CDK9-targeting morpholino or mismatched control sequence and raised to 3 dpf. Embryos with good morpholino uptake were screened, and underwent tailfin transection prior to serial imaging of the wound. (**a**) Representative images of the tailfin from 24 hpi in mismatch [i] or morpholino sequence-injected fish [ii] are shown, with cumulative data (b [i]). n ≥ 38 fish from 4 independent experiments. (**b**) The total neutrophils in the whole embryo at 3 dpf were counted [ii] and the percentage of apoptotic neutrophils at 8 hpi was calculated by quantifying TSA^+^/TUNEL^+^ double positive cells as a percentage of total TSA^+^ cells [iii]. Tg(mpx:EGFP)^i114^ zebrafish eggs were similarly injected with a CDK7-targeting morpholino or mismatched control sequence. (**c**) The tailfin was transected at 3 dpf and imaged at 0, 4 and 24 hpi with cumulative data shown. ≥16 fish per group in 3 independent experiments. *p < 0.05, n/s: not significant, analysed two-way ANOVA followed by post-hoc Newman Keuls test.

The effect of preventing CDK9 protein formation was studied using anti-sense oligonucleotide morpholinos^25^, which prevent the splicing of RNA processing. Western blotting demonstrated successful partial protein knockdown at the concentrations injected (Fig. S1). The fish were then imaged after tailfin transection at 3 dpf, as shown in the example images (Fig. 4a). Morpholino-mediated CDK9-knockdown led to a reduced number of neutrophils at the tailfin 24 h after tailfin wounding, compared to embryos injected with a 5′-mismatch base pair control (represented by Fig. 4a,b[i]). CDK9-morpholino-injected fish had increased numbers of apoptotic neutrophils at 8 hpi in the tailfin (Fig. 4b[iii]). This seemed specific to the injury site, as imaging of the whole embryo revealed no differences in the number of apoptotic neutrophils between mismatch control and CDK9 morpholino groups at all time points (8 hpi and 24 hpi are shown, Fig. S2). Similar to that observed with pharmacological CDK9 inhibition, morpholino-mediated CDK9-knockdown did not affect total fish neutrophils in 3 dpf embryos (Fig. 4b[ii]).

Knockdown of CDK7 was also performed by microinjection of a CDK7-specific morpholino. This revealed no significant difference in neutrophil numbers following tailfin injury with CDK7-knockdown or control groups (Fig. 4c), nor any difference in whole embryo neutrophil numbers (data not shown).

**Figure 5:**
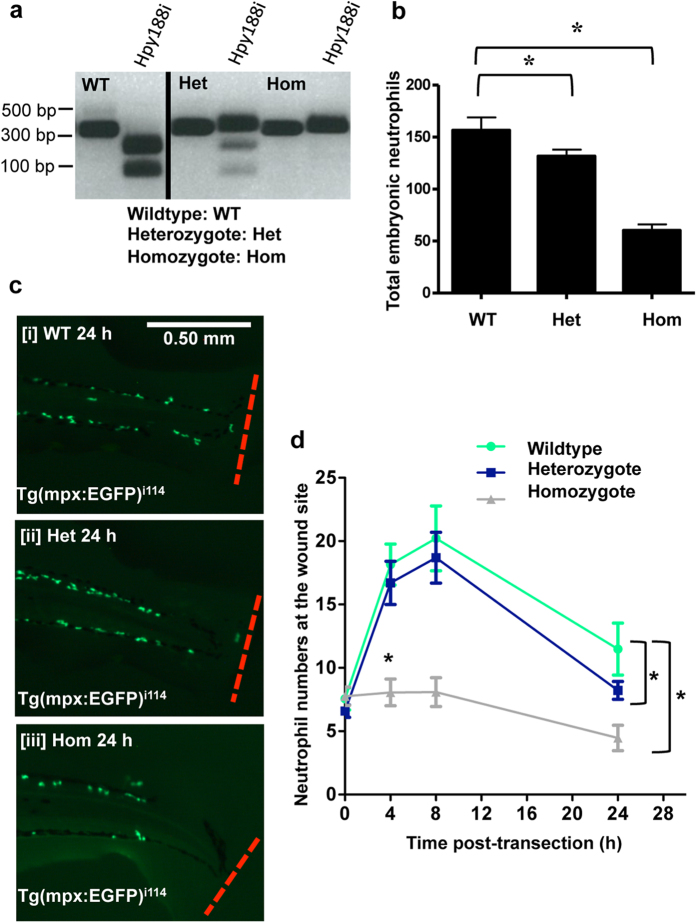
CRISPR/cas9 knockout of CDK9 reduces neutrophilic inflammation. Tg(mpx:EGFP)^i114^ zebrafish eggs were micro-injected with cas9 mRNA and a guide mRNA to target CDK9. (**a**) These animals were raised and in-crossed, then PCR and restriction digests with the restriction enzyme Hpy188i were carried out to assess if embryos were homozygote (Hom), wild type (WT) or heterozygous (Het). The black dividing line is used to show this is two example images cropped from different areas of the same gel. (**b**) Total embryonic neutrophils in fish from each group of embryos were counted. (**c,d**) The tailfin of WT, Hom and Het embryos were transected and the neutrophils at the site of wounding were imaged at various time points and quantified. ≥47 fish per group from 3 independent experiments. Data shown as ±S.E.M. *p < 0.05, n/s: not significant, assessed by two-way ANOVA followed by post-hoc Newman Keuls test.

To further confirm the role of CDK9 in resolution of neutrophilic inflammation, we generated heritable CDK9 knockout Tg(mpx:EGFP)^i114^ fish using a CRISPR/cas9 technique. Homozygote embryos were confirmed as those that had no digestion of a PCR product over the CDK9 region (due to its excision); heterozygotes were confirmed as those that had partial digestion, and wild types had full digestion of the PCR product (Fig. 5a). The DNA sequences of the mutant and a wild type (and the corresponding protein sequence) were analysed (Fig. S3). The total (whole animal) neutrophils at 3 dpf were assessed in homozygote and heterozygote CDK9 mutants, and wild type fish (Fig. 5b). This showed there was a small but significant reduction in total neutrophils between wild type (157 ± 12 cells) and heterozygote (131 ± 6 cells) embryos, and a substantial reduction in homozygote embryos (60 ± 6 cells).

At 3 dpf, tailfin transection was performed on wild type, homozygote and heterozygote zebrafish embryos. The homozygote CDK9 knockout recruited significantly less neutrophils at the wound at 4 and 24 hpi compared with the wild type and heterozygote mutant groups, with 4 ± 1 cells at 24 hpi compared to 11 ± 2 cells in the wild type group (Fig. 5c,d). The heterozygote mutants had similar neutrophil numbers at the wound site at 0 and 4 hpi compared to wild type embryos, but had significantly fewer at 24 hpi, suggesting enhanced resolution of neutrophilic inflammation, similar to that observed with pharmacological CDK9 inhibition (Fig. 2) and CDK9 morpholino knockdown (Fig. 4). Homozygotes had a deformed and reduced body axis and reduced survival compared to heterozygotes and wild type, which were morphologically normal. For this reason, we also calculated the data in respect to the length of the fish (Fig. S4). This further analysis revealed similar differences in neutrophil numbers as observed initially.

### Targeting CDK9 using morpholinos (but not AT7519) delays recruitment of macrophages following wounding

**Figure 6:**
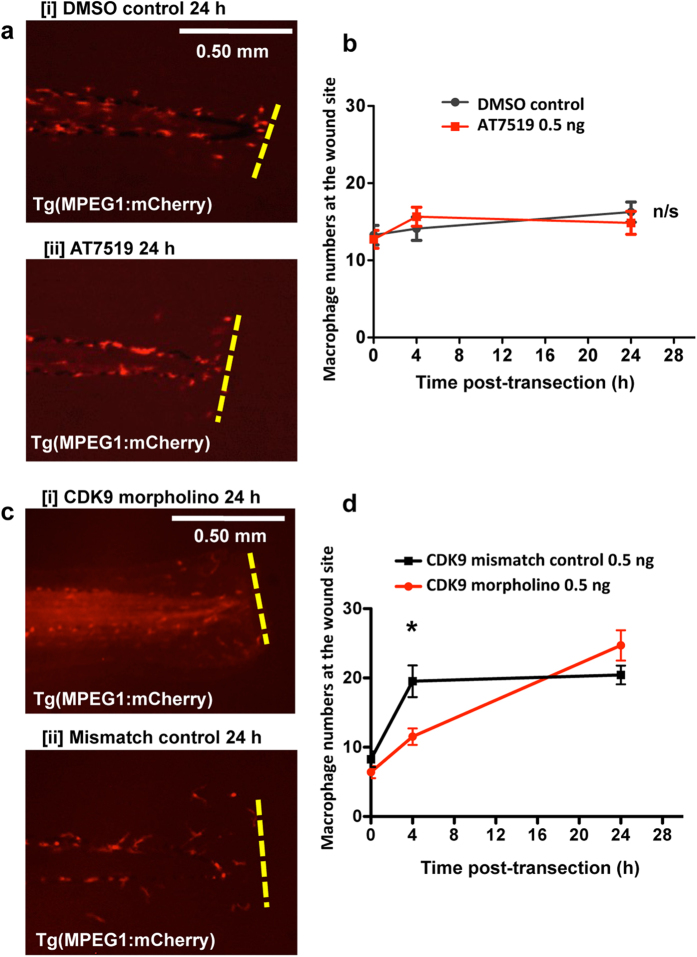
AT7519 and CDK9 morpholino knockdown has no effect on macrophage accumulation post-injury. Tg(MPEG1:mCherry) zebrafish underwent tailfin transection and were treated with DMSO (control) or AT7519 at 4 hpi. (**a**) Example images of DMSO [i] and AT7519 [ii] – treated animals at 24 hpi are shown with cumulative data in (**b**). ≥22 fish per group over 3 independent experiments. Newly laid Tg(MPEG1:mCherry) zebrafish eggs were also injected with a CDK9 splice-blocking morpholino or mismatched control sequence, then raised to 3 dpf. (**c**) The embryo tailfin was transected and the fish imaged at various time points with example images shown at 24 hpi for morpholino [i] and control [ii] fish and cumulative data shown in (**d**). n ≥ 20 fish from 3 independent experiments. Data shown as ±S.E.M. *p < 0.05, n/s: not significant, assessed by two-way ANOVA followed by post-hoc Newman Keuls test.

The effect of CDK9 inhibition on macrophage presence was then assessed. Tg(MPEG1:mCherry) embryos at 3 dpf were injured according to our protocol, then AT7519 was administered at 4 hpi. There was no significant difference (p > 0.05) in macrophage numbers at the wound between DMSO control and AT7519-treated groups (Fig. 6a,b). We then microinjected Tg(MPEG1:mCherry) eggs with the CDK9 morpholino or a mismatched control sequence, and performed the tailfin transection assay at 3 dpf. Macrophage numbers were significantly lower at 4 hpi in the CDK9 morpholino-injected group (12 ± 1 cells), compared to control (20 ± 2 cells); however by 24 h macrophage numbers were not statistically different between both groups (Fig. 6c,d).

### Knockdown of LaRP7 increases neutrophilic inflammation

**Figure 7:**
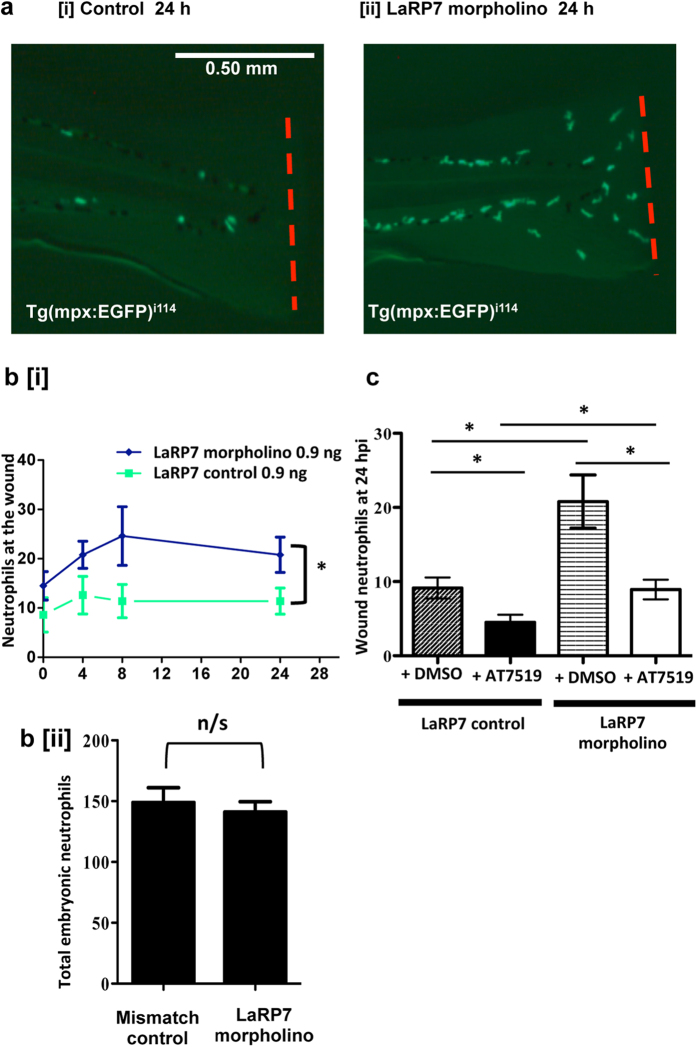
Knockdown of LaRP7 increases inflammation at the wounded tailfin, and this can be inhibited using the CDK inhibitor AT7519. (**a**) Tg(mpx:EFGP)^i114^ zebrafish eggs were injected with a mismatched control or a morpholino sequence to knockdown LaRP7, and at 3 dpf the tailfin was transected with representative images at 24 hpi shown [i, ii]. (**b**) Neutrophils at the wounded tailfin [i] and also in the whole embryo [ii] were quantified. ≥10 fish per group in 3 independent experiments. (**c**) LaRP7 knockdown and control zebrafish were also injected with AT7519/DMSO at 4 hpi and the neutrophils quantified at various time points; shown is the 24 hpi time point. ≥19 fish per group in 3 independent experiments. Data shown as ±S.E.M. *p < 0.05. n/s: not significant. Data analysed by two-way ANOVA followed by post-hoc Newman Keuls or unpaired t-test.

We next examined whether augmenting CDK9 activity would enhance neutrophilic inflammation. To do this, we studied the role of the LaRP7 protein, which forms part of the endogenous P-TEFb inhibitor complex (7SK snRNP). Tg(mpx:EGFP)^i114^ zebrafish eggs were injected with either a LaRP7 morpholino or control sequence, with knockdown confirmed using western blotting (Fig. S1). At 3 dpf, tailfin transection was performed on LaRP7 morpholino- or control sequence-injected embryos. In the LaRP7 morpholino-injected group, there was a significantly increased number of neutrophils observed post-injury (21 ± 4 cells), compared to the control-treated group (10 ± 3, Fig. 7a,7b [i]). This is demonstrated in example images at 24 hpi (Fig. 7a). However, the total neutrophil numbers in the whole embryo were the same between the LaRP7 morpholino and control-injected groups (Fig. 7b[ii]).

AT7519 was injected into LaRP7 morpholino knockdown or control-sequence injected fish at 4 hpi, to examine if CDK inhibition could overcome the effect of increased P-TEFb activity due to LaRP7 knockdown (Fig. 7c). AT7519 significantly (p < 0.05) reduced neutrophil numbers at 24 hpi in the mismatch control injected-group (4 ± 1 neutrophils) and also the LaRP7 morpholino-injected group (9 ± 1 neutrophils). Treatment with AT7519 reduced neutrophil numbers in the LaRP7 morpholino injected fish – numbers of neutrophils were similar to the control group (mismatch sequence + DMSO).

## Discussion

An attractive therapeutic strategy for treatment of acute inflammatory disease is the enhancement of inflammation resolution^1^,^3^. This could be achieved by strategies such as reducing the production of pro-inflammatory mediators, decreasing recruitment of inflammatory granulocytes or promoting the migration of granulocytic cells away from sites of wounding^26^, or promoting local granulocyte apoptosis^27^. It is known that driving granulocyte apoptosis pharmacologically can resolve inflammation in experimental settings *in vivo*^3^,^12^,^13^,^28^. Impaired apoptosis has the opposite effect; for example, inhibition of basal or induced granulocyte apoptosis by caspase inhibitors^12^,^13^ or by a specific inhibitor of pro-apoptotic Bax (V5)^29^ delays inflammation resolution in experimental models of resolving inflammation. A deficiency of tumour necrosis factor-related apoptosis-inducing ligand exacerbates lung injury and fibrosis^30^; and transgenic expression of survival factors such as Bcl-2 in mice extends the lifespan of neutrophils by preventing apoptosis, resulting in severe experimental pneumococcal meningitis, with increased brain inflammation and tissue damage^31^. Similarly, in a mouse model of bacterial sepsis, targeting the pro-apoptotic p53/Puma pathway extends neutrophil survival, prevents resolution and increases mortality rates^32^, suggesting that apoptosis may be essential for effective, non-lethal responses to bacterial infection. Clinical studies also indicate that apoptosis and clearance of apoptotic bodies by macrophages may be aberrant in various inflammatory diseases, including cystic fibrosis and idiopathic pulmonary fibrosis^33^,^34^,^35^.

CDK inhibitor drugs drive neutrophil and eosinophil apoptosis *in vitro* and enhance resolution of inflammation *in vivo*^12^,^13^,^36^,^37^. Previous work from our group found that the flavone compound wogonin, which inhibits CDK9 in cancer cells^38^ can drive neutrophil apoptosis in a zebrafish tailfin wounding model^21^. We have shown here using *in vivo* imaging of zebrafish that pharmacological CDK inhibition at 4 hpi (the peak of the neutrophil response) drives neutrophil apoptosis and enhances resolution of inflammation (Figs 2a–c and 3). This is in keeping with our previous observations where AT7519 induces neutrophil apoptosis *in vitro* and enhances resolution in lung inflammation models in mice^13^. The CDKi AT7519 was more effective at reducing neutrophilic inflammation than flavopiridol, perhaps due to the lower IC50 of AT7519 for CDK9 (<10 nM compared to 20 nM)^39^,^40^. The effect of CDK inhibition appeared specific to inflammatory neutrophils, as total neutrophil numbers were unaffected by AT7519 treatment (Fig. 2d). We would therefore predict that CDK9/P-TEFb activity in neutrophils is enhanced during an inflammatory response, and is therefore more sensitive to inhibition. This hypothesis is supported by our observations showing that RNApol II is enhanced in human neutrophils after exposure to LPS^41^. We have previously shown that human neutrophils express CDK9 and CDK7 which regulate RNA polymerase-II dependent gene transcription, and this may be the mechanism by how CDK inhibitor drugs induce neutrophil apoptosis^41^. However, whether CDK9, CDK7 or combined CDK9/CDK7 activity is responsible for control of neutrophil apoptosis, and which CDK(s) are the target of CDK inhibitor drugs driving inflammation resolution *in vivo* was previously unknown. Here, using genetic targeting, we have defined that CDK9 pathways are pivotal in the resolution of inflammation *in vivo* using a zebrafish model of inflammation.

In the present study, genetic targeting of CDK9 was performed using both morpholino knockdown and CRISPR knockout methods. Mouse knockouts of CDK9, or the CDK9 binding partner cyclin T2, are embryonically lethal^19^,^42^. The use of zebrafish embryos allows us to examine the effect of CDK9 knockout in more detail, as zebrafish possess the ability to survive without a functioning cardiovascular system in the first days of life, allowing the study of embryonically lethal phenotypes^43^. In addition, it is very difficult to effectively knockdown genes in primary human neutrophils, which do not proliferate and have a short lifespan.

Targeting CDK9 using morpholinos reduced neutrophil inflammation post-wounding (Fig. 4B[i]), despite no effect on total neutrophil numbers (Fig. 4b[ii]). The neutrophil numbers at the peak of inflammation were similar in control and CDK9 morpholino-treated fish, but at 24 hpi there were less neutrophils in the CDK9 morpholino knockdown animals; suggesting that the effect of CDK9 inhibition is more pronounced during the later ‘resolution’ phase. In AT7519-treated and CDK9 morpholino embryos, we observed that the neutrophils appeared to be smaller and had a more rounded morphology, commonly observed during apoptosis (Fig. 2a,b). TUNEL staining revealed there was an increased apoptosis of neutrophils: this was noted between 8–12 hpi after the tailfin assay was conducted (Figs 3b and 4B[iii]), resulting in the phenotype of reduced tailfin neutrophils by 24 hpi. It is unclear why there were not increased apoptosis levels in the drug treated group at 24 h, but this can perhaps be attributed to loss of drug efficacy over time. It may well be the case that most neutrophils which would be recruited have undergone apoptosis and been cleared by this time point.

Macrophage recruitment after CDK9 knockdown was initially delayed at 4 hpi in the CDK9-morpholino animals, compared to control, but restored to normal levels by 24 hpi (Fig. 6c,d). This initial delay could perhaps be secondary to reduced production of pro-inflammatory mediators such as TNF, which require CDK9 for transcription^17^. CDK9 inhibition is also thought to affect expression of adhesion molecules (such as ICAM-1) required for leukocytes to migrate through the endothelium during inflammatory responses^44^. We have seen no evidence of *in situ* proliferation of macrophages during time lapse imaging, suggesting most of the increase in macrophage numbers by 24 hpi is due to enhanced migration. Pharmacological CDK inhibition using AT7519 did not affect macrophage numbers post-wounding (Fig. 6b). Previous work has shown that pharmacological CDK inhibition using AT7519 only transiently affects macrophage numbers in an LPS-induced lung inflammation model in mice, and increases the numbers of macrophages with apoptotic bodies present in the bronchiolar lavage^13^. It is desirable not to affect macrophages detrimentally during the resolution phase, as they are required for clearance of apoptotic neutrophils. In the future, it would be interesting to study if there is increased uptake of apoptotic neutrophils by each macrophage, in order to respond to enhanced apoptosis, as macrophage numbers appear unaffected by the drug treatment.

The homozygote CDK9 knockout embryos possessed an abnormal morphology, making them less useful for the purpose of studying inflammation; they had a reduced total number of neutrophils, as well as a reduced number of neutrophils at the wound (Fig. 5b,d). In contrast, the heterozygote knockout embryos were morphologically healthy. There was a reduction in the number of neutrophils at the injury site at 24 hpi in both homozygote and heterozygote knockout groups. Taken together with the morpholino data, these genetic data highlight the importance of CDK9 in resolution of neutrophilic inflammation. A similar effect was seen with pharmacological CDK9 inhibition, and whilst the actions of the genetic or pharmacological intervention are different, the targets are the same and the impact was observed in the resolution of the inflammatory response. Morpholino-knockdown of another component of this pathway, CDK7, had no effect on the neutrophil response post-wounding. It has been shown in cell lines that CDK7 and CDK9 may have overlapping activity for phosphorylation of serine 5 on RNA polymerase II, and this may explain why loss of CDK7 may not result in a phenotype^45^. It is also possible that CDK7 is not such a crucial part of the P-TEFb complex in zebrafish; previous work on CDK7 in zebrafish has focused on its role in embryogenesis and zebrafish development^46^. In the future, the role of CDK7 could be better examined by creating a CDK7-knockout zebrafish, or by testing a CDK7-specific pharmacological inhibitor.

Although outwith the scope of the current manuscript, the effect of CDK9 inhibition on transcription of inflammatory mediators is an area of future study. The role of cytokines and chemokines in the zebrafish model is a developing field, and other novel chemoattractants have been described in zebrafish such as hydrogen peroxide, which may also be important in our model system^47^. The effect of CDK9 manipulation on these mediators will help us define the role of CDK9 in neutrophil migration, i.e. whether knocking down CDK9 affects cellular recruitment as well as neutrophil apoptosis at the injury site. It is known in zebrafish that reverse migration of neutrophils away from sites of inflammation occurs^48^,^49^,^50^, a cellular behaviour also thought to occur in mammals^51^. It will also therefore be interesting to investigate if CDK9 is a regulator of reverse neutrophil migration, perhaps via the regulation of specific molecules known to be important in reverse migration^52^. Interestingly, zebrafish neutrophil migration away from sites of wounding can be enhanced pharmacologically by treatment with the compound Tanshinone IIA, a derivative of the Chinese medicinal herb *Salvia miltiorrhiza*^26^,^49^. This may be another mechanism by which resolution of inflammation could be enhanced.

In the future, we would also like to investigate the effect of targeting CDK9 on tissue regeneration in this model, and what effect targeting neutrophils in this way affects regeneration. It has been suggested that macrophages rather than neutrophils are most important for regeneration^53^,^54^.

A previous study investigating the role of CDK9 in heart laser injury showed that inhibition of the endogenous P-TEFb inhibitor, LaRP7, rescued the phenotype of embryos in which CDK9 was knocked down^55^. The same study showed that CDK9 knockdown reduces cardiomyocyte proliferation, whereas LaRP7 knockdown increases it. In the tailfin injury assay used here, LaRP7 knockdown had the opposite effect to knockdown of CDK9 on neutrophil numbers at the wound site, namely by increasing neutrophil recruitment to the site of wounding (Fig. 7a,b). Micro-injection with AT7519 returned neutrophil numbers at inflammatory sites to normal levels, indicating CDK inhibition can overcome increased P-TEFb activity (Fig. 7c). This indicates that CDK9 inhibitors could be useful therapeutic agents for enhancing the resolution of inflammation.

CDK inhibitors (including AT7519) have already been tested successfully in clinical trials as potential cancer therapeutics and are well tolerated^56^,^57^. Neutrophils highly express the pro-survival protein Mcl-1, which is transcribed by CDK9 activity, and they rely on it for survival; unlike other cells such as macrophages^12^,^13^. Previous studies have utilised local administration of CDKi compounds (e.g. intra-tracheal administration to the lung^13^). In this way, systemic side-effects could potentially be minimised. Hence, CDK9 may be a good and specific target for neutrophil apoptosis, either as a short course of treatment in acute neutrophilic inflammation (e.g. acute respiratory distress syndrome), or for chronic diseases characterised by episodes of acute inflammation (e.g. rheumatoid arthritis). Previous studies have also shown that timely neutrophil apoptosis could be crucial in host defence in bacterial infections^31^,^32^. As such, CDK9 inhibitor drugs could well be a feasible strategy in the clinic for treatment of inflammatory diseases.

## Methods and Materials

### Zebrafish Husbandry

All experiments were approved and carried out in a UK Home Office approved facility at the University of Edinburgh under a UK Home Office Project Licence, and in accordance with the accepted standards of humane animal care under the regulation of the Animal (Scientific Procedures) Act UK 1986 and EU Directive 2010/63/EU. All animals used in these experimental studies were under the age of 5 days post fertilisation. The following transgenic zebrafish lines were used: Tg(mpx:EGFP)^i114^ and Tg(MPEG1:mCherry)^58^,^59^. The embryos were housed at 28.5 °C and imaged at room temperature (22 °C).

### Experimental Design

The aim of the study was to examine if different methods to inhibit CDK9 impacted on resolution of neutrophil and macrophage inflammatory responses *in vivo*, using Tg(mpx:EGFP)^i114^ and Tg(MPEG1:mCherry) zebrafish with labelled neutrophils and macrophages. CDK9-knockout mutants were also created using CRISPR/cas9. The median tailfin of zebrafish embryos at 3 days post fertilisation (3 dpf) was transected using a sterile scalpel, avoiding damage to the body and vasculature (depicted in Fig. 1a)^21^,^60^. Each embryo was then dispensed in a single well of a 48 well plate. The embryos were either treated with a CDK inhibitor drug from 4 hours post-injury (hpi), or had been pre-injected with morpholino sequences to knockdown CDK9, CDK7 or LaRP7. The embryos were imaged at various hpi (e.g. 0, 4, 24 hpi). Each individual experiment was repeated on 3 separate occasions.

### Imaging

Transected embryos were then imaged on a Leica MZ 16 F stereomicroscope with EL6000 fluorescent light source, or confocal microscope (Leica sp5) at various time points after transection. Immediately after injury (0 hpi) indicates zebrafish that have been transected and then immediately imaged. The EGFP^+^ neutrophils were visualised by excitation at 480 nm, mCherry macrophages at 587 nm, and TUNEL stained apoptotic DNA at 570 nm. A standardised area was selected for analysis that was applied to every image taken using ImageJ, depicted in Fig. 1a. This corresponds to a length of 0.5 mm from the tip of the body of the fish^21^. The red line depicts the line of transection. For CDK9 knockout fish neutrophil recruitment experiments, the fish length was measured from scale and the neutrophils recruited per mm were calculated. Time-lapse imaging was performed of the injured tailfin. Images of the injured tailfin were taken every 5 min and the videos were analysed using ImageJ to track cells.

### Microinjection of Morpholinos and CRISPR/cas9 RNA

Microinjection was carried out as described^25^. Fertilised eggs (1–4 cell stage) were injected using an IM300 microinjector (Narishige) with 1 nL of morpholino or RNA. The following amounts were injected: 0.5 ng of CDK9 (splice-blocking or control); 1.8 ng of CDK7 (splice-blocking or control) or 0.9 ng of LaRP7 (splice-blocking or control). The following splice-blocking morpholinos (Gene Tools LLC) were designed to target the following genes (Table 1). The CDK9 and LaRP7 morpholinos have been successfully used in a previous study^55^.

### Microinjection of AT7519 into the yolk sac of the zebrafish

0.5 ng of AT7519 (a kind gift from Astex Pharmaceuticals) was micro-injected into the yolk sac of 3 dpf zebrafish at 4 h following tailfin transection.

### Incubation of zebrafish with flavopiridol

The tailfin of zebrafish at 3 dpf were transected and at 4 hpi, each embryo was added to an individual well of a 48 well plate containing 500 μL of embryo medium with 1 μM of flavopiridol or ≤1% dimethyl sulfoxide vehicle control (DMSO, both Sigma Aldrich). The fish were serially imaged, to determine neutrophilic inflammation.

### Live Imaging

Using a Leica M2 16 F fluorescent stereomicroscope, the embryos were imaged in system water (0.8 g sodium bicarbonate, 4.5 mL Marine Salts, 750 L H_2_0, 0.6 mL methylthioninium chloride [methylene blue]) with 4.2% (v/v) Tricaine to induce anaesthesia. Images were taken at 40 x, 80 x or 100 x magnification using a Leica DFC300 FX Digital Colour Camera connected to LAS AF (Leica) V3 software connected to the Leica MZ 16 F microscope. For confocal imaging (Leica sp5), the embryos were mounted in 1.5% low melting-point agarose (Sigma Aldrich).

### Western blotting

Individual fish were lysed in 30 μL of RIPA buffer with protease cocktail inhibitor (Sigma Aldrich) at 3dpf. Western blotting was performed and the blots probed with antibodies against CDK9 or CDK7 (Santa Cruz Biotechnology) and LaRP7 (Abcam), all used at 1:1000. Appropriate secondary HRP-conjugated antibodies were used (Dako, Cambridgeshire UK) and developed by chemi-luminescence (Amersham).

### TUNEL Staining

Embryos were fixed in 4% PFA then stored in methanol. Following rehydration, the GFP signal was enhanced using a Tyrosine Signal Amplification kit (Perkin Elmer). TUNEL staining was then performed using ApopTag Red *In Situ* kit (Millipore) to label apoptotic cells, as described^26^. Imaging of embryos was also carried out using confocal microscopy of embryos mounted in 1.5% agarose.

### CRISPR guide RNA generation

The use of the CRISPR/cas9 for gene editing is described in zebrafish^61^,^62^. The gRNA guide oligonucleotide sequences were designed with an online CRISPR Design Tool ( http://www.genome-engineering.org/crispr/?page_id=41). From this, guide sequence templates were synthesised (Eurofins) then annealed (5′–3′): ATAGTGAGTCGTATTA and the T7 primer TAATACGACTCACTATAG. The MEGAscript T7 kit was used to transcribe the guide RNA *in vitro* and the MEGAclear kit was used to further purify the RNA. The cas9 *in vitro* transcription was performed as previously described^62^. The Cas9 was linearized using *Not*I, then transcribed *in vitro* using the SP6 mMESSAGE mMACHINE kit. The guide and cas9 mRNA were co-microinjected into the single cell of newly laid zebrafish eggs. The fish were raised and crossed with Tg(mpx:EGFP)^i114^ embryos, and this generation was used to create F2 embryos for experiments. Following experimentation, the embryos were genotyped by performing PCR over the CDK9 region then digesting with the restriction enzyme Hpy188I (which targeted a region of DNA present within the guide region of the CRISPR RNA).

### Analysis, Graphing & Statistics

Following acquisition, using LAS AF (Leica) V3 software, images were processed using ImageJ (Fiji) software (National Institutes of Health, Bethesda). Graphs and statistical analysis were created on GraphPad Prism 5 software. Data were analysed by two-way or one-way ANOVA followed by Newman-Keuls multiple comparison post hoc test, or where appropriate, by using an unpaired t-test. *indicates p ≤ 0.05.

## Additional Information

**How to cite this article**: Hoodless, L. J. *et al*. Genetic and pharmacological inhibition of CDK9 drives neutrophil apoptosis to resolve inflammation in zebrafish *in vivo*. *Sci. Rep.* **6**, 36980; doi: 10.1038/srep36980 (2016).

**Publisher’s note:** Springer Nature remains neutral with regard to jurisdictional claims in published maps and institutional affiliations.

## Supplementary Material

Supplementary Information

## Figures and Tables

**Table 1:** Morpholino sequences used in the study, and ENSEMBL ID of genes of interest.

Gene	ENSEMBL ID	Morpholino sequence 5′–3′
CDK9	ENSDARG00000044811	Splice-blocking morpholinos:
		(*a*) GTAAAATATTCGTACTTTTCACCGC
		(*b*) GGTGCATTTTCTTACCCCTTCTTTC
		Mismatch: GGTCCATTTTGTTAGCCGTTGTTTC
CDK7	ENSDARG00000051916	Splice-blocking morpholino:
		ATAAAGTGTTTCTTTACCCTGTTCA
		Mismatch: ATATACTGTTTGTTTACCGTCTTCA
LaRP7	ENSDARG00000017315	Splice-blocking morpholino:
		TCATCTCCATACTAAACCAAACTGT
		Mismatch: TGATGTCCATAGTAAACGAAACTCT

## References

[b1] Duffin R., Leitch A. E., Fox S., Haslett C. & Rossi A. G. Targeting granulocyte apoptosis: mechanisms, models, and therapies. Immunol. Rev . 236, 28–40 (2010). 20636806 10.1111/j.1600-065X.2010.00922.x

[b2] Jones H. R., Robb C. T., Perretti M. & Rossi A. G. The role of neutrophils in inflammation resolution. Semin. Immunol , 10.1016/j.smim.2016.03.007 (2016). 27021499

[b3] Leitch A. E., Duffin R., Haslett C. & Rossi A. G. Relevance of granulocyte apoptosis to resolution of inflammation at the respiratory mucosa. Mucosal Immunol 1, 350–363 (2008). 19079199 10.1038/mi.2008.31 PMC7102379

[b4] Soehnlein O. Multiple roles for neutrophils in atherosclerosis. Circ. Res. 110, 875–888 (2012). 22427325 10.1161/CIRCRESAHA.111.257535

[b5] Zenaro E. . Neutrophils promote Alzheimer’s disease-like pathology and cognitive decline via LFA-1 integrin. Nat. Med . 21, 880–886 (2015). 26214837 10.1038/nm.3913

[b6] Feng Y., Renshaw S. & Martin P. Live imaging of tumor initiation in zebrafish larvae reveals a trophic role for leukocyte-derived PGE₂. Curr. Biol. 22, 1253–1259 (2012). 22658594 10.1016/j.cub.2012.05.010 PMC3398414

[b7] Lahoz-Beneytez J. . Human neutrophil kinetics: modeling of stable isotope labeling data supports short blood neutrophil half-lives. Blood 127, 3431–3438 (2016). 27136946 10.1182/blood-2016-03-700336 PMC4929930

[b8] Kolaczkowska E. & Kubes P. Neutrophil recruitment and function in health and inflammation. Nat. Rev. Immunol. 13, 159–175 (2013). 23435331 10.1038/nri3399

[b9] Fox S., Leitch A. E., Duffin R., Haslett C. & Rossi A. G. Neutrophil apoptosis: relevance to the innate immune response and inflammatory disease. J Innate Immun 2, 216–227 (2010). 20375550 10.1159/000284367 PMC2956014

[b10] Muñoz L. E., Lauber K., Schiller M., Manfredi A. A. & Herrmann M. The role of defective clearance of apoptotic cells in systemic autoimmunity. Nat Rev Rheumatol 6, 280–289 (2010). 20431553 10.1038/nrrheum.2010.46

[b11] McKeon D. J. . Prolonged survival of neutrophils from patients with Delta F508 CFTR mutations. Thorax 63, 660–661 (2008). 18587042 10.1136/thx.2008.096834

[b12] Rossi A. G. . Cyclin-dependent kinase inhibitors enhance the resolution of inflammation by promoting inflammatory cell apoptosis. Nat. Med . 12, 1056–1064 (2006). 16951685 10.1038/nm1468

[b13] Lucas C. D. . Downregulation of Mcl-1 has anti-inflammatory pro-resolution effects and enhances bacterial clearance from the lung. Mucosal Immunol 7, 857–868 (2014). 24280938 10.1038/mi.2013.102 PMC3940382

[b14] Leitch A. E. . The cyclin-dependent kinase inhibitor R-roscovitine down-regulates Mcl-1 to override pro-inflammatory signalling and drive neutrophil apoptosis. Eur. J. Immunol. 40, 1127–1138 (2010). 20127676 10.1002/eji.200939664

[b15] Canduri F., Perez P. C., Caceres R. A. & de Azevedo W. F. CDK9 a potential target for drug development. Med Chem 4, 210–218 (2008). 18473913 10.2174/157340608784325205

[b16] MacCallum D. E. . Seliciclib (CYC202, R-Roscovitine) induces cell death in multiple myeloma cells by inhibition of RNA polymerase II-dependent transcription and down-regulation of Mcl-1. Cancer Res . 65, 5399–5407 (2005). 15958589 10.1158/0008-5472.CAN-05-0233

[b17] Smallie T. . IL-10 inhibits transcription elongation of the human TNF gene in primary macrophages. J. Exp. Med. 207, 2081–2088 (2010). 20805562 10.1084/jem.20100414 PMC2947066

[b18] Barboric M. . 7SK snRNP/P-TEFb couples transcription elongation with alternative splicing and is essential for vertebrate development. Proc. Natl. Acad. Sci. USA 106, 7798–7803 (2009). 19416841 10.1073/pnas.0903188106 PMC2683122

[b19] Kohoutek J. . Cyclin T2 is essential for mouse embryogenesis. Mol. Cell. Biol. 29, 3280–3285 (2009). 19364821 10.1128/MCB.00172-09 PMC2698739

[b20] Henry K. M., Loynes C. A., Whyte M. K. B. & Renshaw S. A. Zebrafish as a model for the study of neutrophil biology. J. Leukoc. Biol. 94, 633–642 (2013). 23463724 10.1189/jlb.1112594

[b21] Lucas C. D. . Flavones induce neutrophil apoptosis by down-regulation of Mcl-1 via a proteasomal-dependent pathway. FASEB J . 27, 1084–1094 (2013). 23195034 10.1096/fj.12-218990 PMC3574292

[b22] Loynes C. A. . Pivotal Advance: Pharmacological manipulation of inflammation resolution during spontaneously resolving tissue neutrophilia in the zebrafish. J. Leukoc. Biol. 87, 203–212 (2010). 19850882 10.1189/jlb.0409255 PMC2812557

[b23] Chao S. H. . Flavopiridol inhibits P-TEFb and blocks HIV-1 replication. Journal of Biological Chemistry 275, 28345–28348 (2000). 10906320 10.1074/jbc.C000446200

[b24] Santo L. . AT7519, A novel small molecule multi-cyclin-dependent kinase inhibitor, induces apoptosis in multiple myeloma via GSK-3beta activation and RNA polymerase II inhibition. Oncogene 29, 2325–2336 (2010). 20101221 10.1038/onc.2009.510 PMC3183744

[b25] Moulton J. D. Using morpholinos to control gene expression. Curr Protoc Nucleic Acid Chem **Chapter 4,** Unit 4.30–4.30.24 (2007). 10.1002/0471142700.nc0430s27 PMC7162184 18428977

[b26] Robertson A. L. . A zebrafish compound screen reveals modulation of neutrophil reverse migration as an anti-inflammatory mechanism. Sci Transl Med 6, 225ra29 (2014). 10.1126/scitranslmed.3007672 PMC4247228 24574340

[b27] Ortega-Gómez A., Perretti M. & Soehnlein O. Resolution of inflammation: an integrated view. EMBO Mol Med 5, 661–674 (2013). 23592557 10.1002/emmm.201202382 PMC3662311

[b28] Vandivier R. W., Henson P. M. & Douglas I. S. Burying the dead: the impact of failed apoptotic cell removal (efferocytosis) on chronic inflammatory lung disease. Chest 129, 1673–1682 (2006). 16778289 10.1378/chest.129.6.1673

[b29] Sawatzky D. A., Willoughby D. A., Colville-Nash P. R. & Rossi A. G. The involvement of the apoptosis-modulating proteins ERK 1/2, Bcl-xL and Bax in the resolution of acute inflammation *in vivo*. Am. J. Pathol. 168, 33–41 (2006). 16400007 10.2353/ajpath.2006.050058 PMC1592663

[b30] McGrath E. E. . Deficiency of tumour necrosis factor-related apoptosis-inducing ligand exacerbates lung injury and fibrosis. Thorax 67, 796–803 (2012). 22496351 10.1136/thoraxjnl-2011-200863 PMC3426075

[b31] Koedel U. . Apoptosis Is essential for neutrophil functional shutdown and determines tissue damage in experimental pneumococcal meningitis. PLoS Pathog 5, e1000461–13 (2009). 19478887 10.1371/journal.ppat.1000461 PMC2682662

[b32] Garrison S. P. . The p53-target gene puma drives neutrophil-mediated protection against lethal bacterial sepsis. PLoS Pathog 6, e1001240 (2010). 21203486 10.1371/journal.ppat.1001240 PMC3009602

[b33] Poon I. K. H., Lucas C. D., Rossi A. G. & Ravichandran K. S. Apoptotic cell clearance: basic biology and therapeutic potential. Nat. Rev. Immunol. 14, 166–180 (2014). 24481336 10.1038/nri3607 PMC4040260

[b34] Vandivier R. W. . Impaired clearance of apoptotic cells from cystic fibrosis airways. Chest 121, 89S (2002). 10.1378/chest.121.3_suppl.89s 11893715

[b35] Morimoto K., Janssen W. J. & Terada M. Defective efferocytosis by alveolar macrophages in IPF patients. Respir Med 106, 1800–1803 (2012). 22999220 10.1016/j.rmed.2012.08.020 PMC4030720

[b36] Alessandri A. L. . Induction of eosinophil apoptosis by the cyclin-dependent kinase inhibitor AT7519 promotes the resolution of eosinophil-dominant allergic inflammation. PLoS ONE 6, e25683 (2011). 21984938 10.1371/journal.pone.0025683 PMC3184151

[b37] Lucas C. D. . Wogonin induces eosinophil apoptosis and attenuates allergic airway inflammation. Am. J. Respir. Crit. Care Med. 191, 626–636 (2015). 25629436 10.1164/rccm.201408-1565OC PMC4384778

[b38] Polier G. . Wogonin and related natural flavones are inhibitors of CDK9 that induce apoptosis in cancer cells by transcriptional suppression of Mcl-1. Cell Death Dis 2, e182 (2011). 21776020 10.1038/cddis.2011.66 PMC3199715

[b39] Squires M. S. . Biological characterization of AT7519, a small-molecule inhibitor of cyclin-dependent kinases, in human tumor cell lines. Mol. Cancer Ther. 8, 324–332 (2009). 19174555 10.1158/1535-7163.MCT-08-0890

[b40] Chao S. H. & Price D. H. Flavopiridol inactivates P-TEFb and blocks most RNA polymerase II transcription *in vivo*. Journal of Biological Chemistry 276, 31793–31799 (2001). 11431468 10.1074/jbc.M102306200

[b41] Leitch A. E. . Cyclin-dependent kinases 7 and 9 specifically regulate neutrophil transcription and their inhibition drives apoptosis to promote resolution of inflammation. Cell Death Differ . 19, 1950–1961 (2012). 22743999 10.1038/cdd.2012.80 PMC3504709

[b42] Shim E. Y., Walker A. K., Shi Y. & Blackwell T. K. CDK-9/cyclin T (P-TEFb) is required in two postinitiation pathways for transcription in the *C. elegans* embryo. Genes Dev . 16, 2135–2146 (2002). 12183367 10.1101/gad.999002 PMC186450

[b43] Chen J. N. . Left-right pattern of cardiac BMP4 may drive asymmetry of the heart in zebrafish. Development 124, 4373–4382 (1997). 9334285 10.1242/dev.124.21.4373

[b44] Berberich N. . Roscovitine blocks leukocyte extravasation by inhibition of cyclin-dependent kinases 5 and 9. Br. J. Pharmacol. 163, 1086–1098 (2011). 21391976 10.1111/j.1476-5381.2011.01309.x PMC3130954

[b45] Glover-Cutter K. . TFIIH-associated Cdk7 kinase functions in phosphorylation of C-terminal domain Ser7 residues, promoter-proximal pausing, and termination by RNA polymerase II. Mol. Cell. Biol. 29, 5455–5464 (2009). 19667075 10.1128/MCB.00637-09 PMC2756882

[b46] Liu Q. Y., Wu Z. L., Lv W. J., Yan Y. C. & Li Y. P. Developmental expression of Cyclin H and Cdk7 in zebrafish: the essential role of Cyclin H during early embryo development. Cell Res 17, 163–173 (2007). 17287831 10.1038/sj.cr.7310144

[b47] Niethammer P., Grabher C., Look A. T. & Mitchison T. J. A tissue-scale gradient of hydrogen peroxide mediates rapid wound detection in zebrafish. Nature 459, 996–999 (2009). 19494811 10.1038/nature08119 PMC2803098

[b48] Starnes T. W. & Huttenlocher A. Neutrophil reverse migration becomes transparent with zebrafish. Adv Hematol 2012, 398640–11 (2012). 22844288 10.1155/2012/398640 PMC3401556

[b49] Lucas C. D., Hoodless L. J. & Rossi A. G. Swimming against the tide: drugs drive neutrophil reverse migration. Sci Transl Med 6, 225fs9 (2014). 10.1126/scitranslmed.3008666 24574338

[b50] Brown S. B. . Class III antiarrhythmic methanesulfonanilides inhibit leukocyte recruitment in zebrafish. J. Leukoc. Biol. 82, 79–84 (2007). 17431092 10.1189/jlb.0107030

[b51] Colom B. . Leukotriene B4-neutrophil elastase axis drives neutrophil reverse transendothelial cell migration *in vivo*. Immunity 42, 1075–1086 (2015). 26047922 10.1016/j.immuni.2015.05.010 PMC4504024

[b52] Woodfin A. . The junctional adhesion molecule JAM-C regulates polarized transendothelial migration of neutrophils *in vivo*. Nat. Immunol. 12, 761–769 (2011). 21706006 10.1038/ni.2062 PMC3145149

[b53] Li L., Yan B., Shi Y.-Q., Zhang W.-Q. & Wen Z.-L. Live imaging reveals differing roles of macrophages and neutrophils during zebrafish tail fin regeneration. J. Biol. Chem. 287, 25353–25360 (2012). 22573321 10.1074/jbc.M112.349126 PMC3408142

[b54] Petrie T. A. . Macrophages modulate adult zebrafish tail fin regeneration. Development 141, 2581–2591 (2014). 24961798 10.1242/dev.098459 PMC4067955

[b55] Matrone G. . CDK9 and its repressor LARP7 modulate cardiomyocyte proliferation and response to injury in the zebrafish heart. J. Cell. Sci , 10.1242/jcs.175018 (2015). PMC4696495 26542022

[b56] Chen E. X. . A Phase I study of cyclin-dependent kinase inhibitor, AT7519, in patients with advanced cancer: NCIC Clinical Trials Group IND 177. Br. J. Cancer 111, 2262–2267 (2014). 25393368 10.1038/bjc.2014.565 PMC4264455

[b57] Mahadevan D. . A phase I pharmacokinetic and pharmacodynamic study of AT7519, a cyclin-dependent kinase inhibitor in patients with refractory solid tumors. Ann. Oncol. 22, 2137–2143 (2011). 21325451 10.1093/annonc/mdq734

[b58] Renshaw S. A. . A transgenic zebrafish model of neutrophilic inflammation. Blood 108, 3976–3978 (2006). 16926288 10.1182/blood-2006-05-024075

[b59] Ellett F., Pase L., Hayman J. W., Andrianopoulos A. & Lieschke G. J. mpeg1 promoter transgenes direct macrophage-lineage expression in zebrafish. Blood 117, e49–e56 (2011). 21084707 10.1182/blood-2010-10-314120 PMC3056479

[b60] Hoodless L. J., Robb C. T., Felton J. M., Tucker C. S. & Rossi A. G. In Laser Capture Microdissection (ed. Murray G. I. ) 1336, 179–209 (Springer: New York, , 2016).

[b61] Sander J. D. & Joung J. K. CRISPR-Cas systems for editing, regulating and targeting genomes. Nature Biotechnology 32, 347–355 (2014). 10.1038/nbt.2842 PMC4022601 24584096

[b62] Hruscha A. . Efficient CRISPR/Cas9 genome editing with low off-target effects in zebrafish. Development 140, 4982–4987 (2013). 24257628 10.1242/dev.099085

