# Supplementary material for: Genetic and pharmacological inhibition of CDK9 drives neutrophil apoptosis to resolve inflammation in zebrafish in vivo
Source: Sci Rep. 2016 Nov 11;5:36980. doi: 10.1038/srep36980 (PMC5105078; doi:10.1038/srep36980)
Supplement: Supplementary Information [file srep36980-s1.doc]

**SUPPLEMENTARY FIGURES**

**Title: Genetic and pharmacological inhibition of CDK9 drives neutrophil apoptosis to resolve inflammation in zebrafish *in vivo***

**One Sentence Summary:** Targeting CDK9 pharmacologically and genetically *in vivo* drives neutrophil apoptosis in a zebrafish tailfin wounding model, showing resolution of inflammation can be enhanced by inhibition of CDK9.

**Authors:** Laura J. Hoodless^1^, Christopher D. Lucas^1^, Rodger Duffin^1^, Martin A. Denvir^2^, Christopher Haslett^1^, Carl S. Tucker^2^, Adriano G. Rossi^1^*

**Affiliations:**

**^1^**MRC Centre for Inflammation Research, The Queen’s Medical Research Institute, The University of Edinburgh, Edinburgh, United Kingdom. EH16 4TJ

**^2^**BHF Centre for Cardiovascular Science, The Queen’s Medical Research Institute, The University of Edinburgh, Edinburgh, United Kingdom. EH16 4TJ

*Corresponding author: a.g.rossi@ed.ac.uk

**
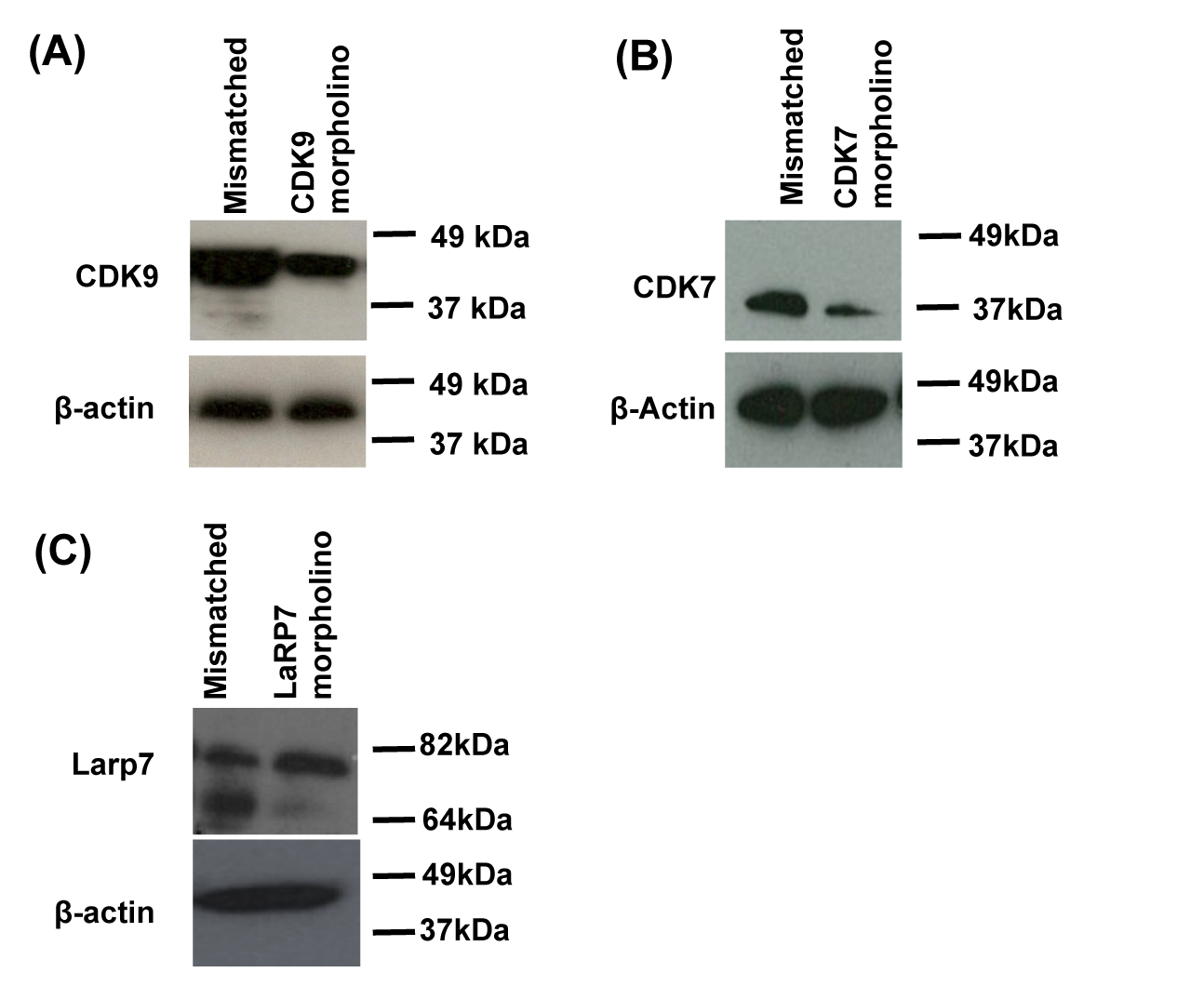
**

**Fig. S1. Western blotting to assess protein reduction using morpholino knockdown**

The knockdown of genes using morpholinos was assessed using western blotting and appropriate antibodies in CDK9 knockdown (43 kDa, A), CDK7 knockdown (42 kDa, B) and LaRP7 knockdown (67 kDa, C) zebrafish (or mismatch-injected controls). Shown here are cropped examples from images of gels.


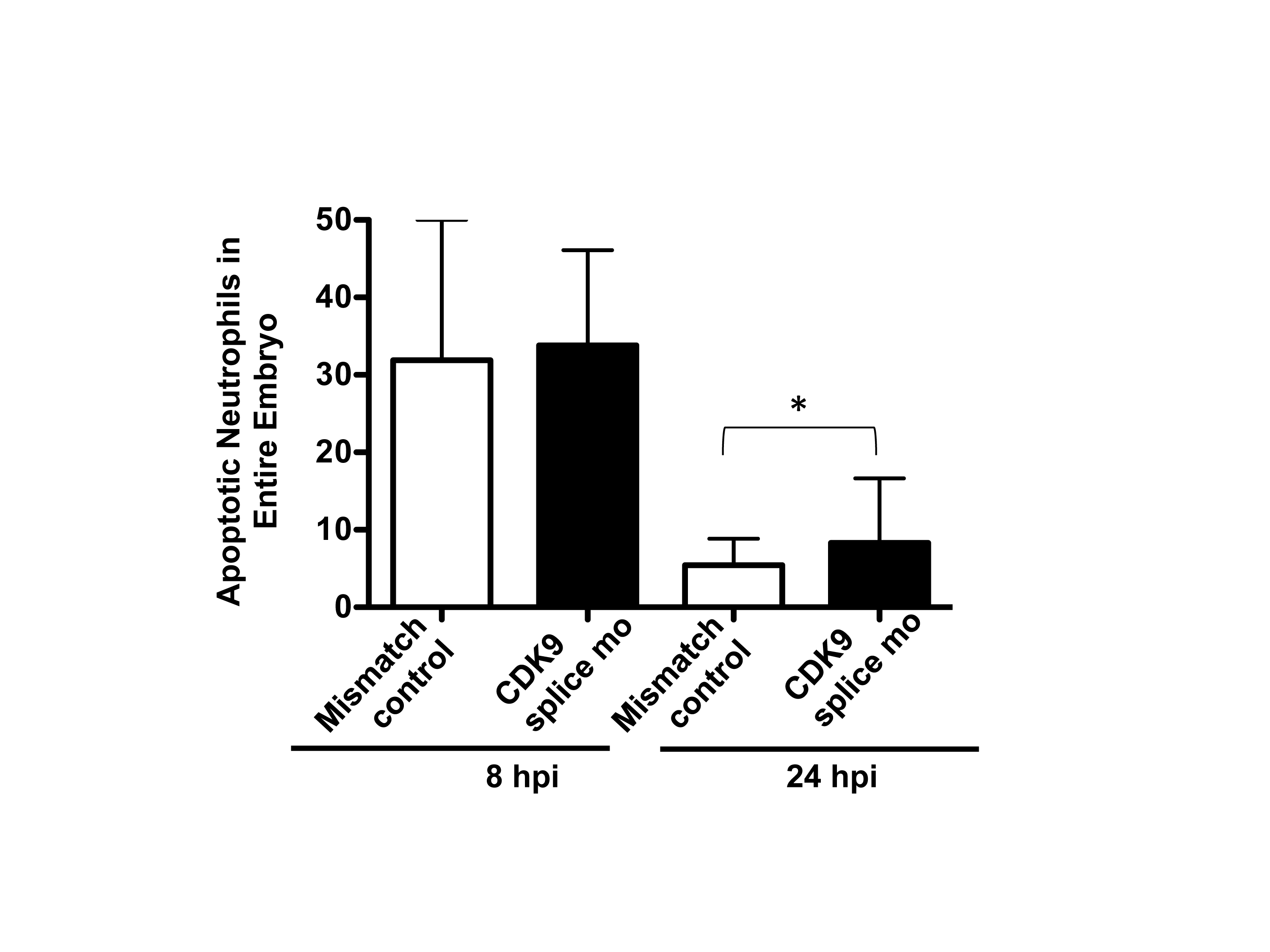


**Fig. S2. Apoptotic cells in the whole embryo in fish injected with CDK9 splice-blocking morpholinos or a mismatch control.**

At 3 dpf the morpholino- or control-injected fish were fixed at various time points post-injury and TSA/TUNEL staining and imaging of the whole embryo performed. Neutrophils with apoptotic nuclei (in the whole fish) were counted.

**
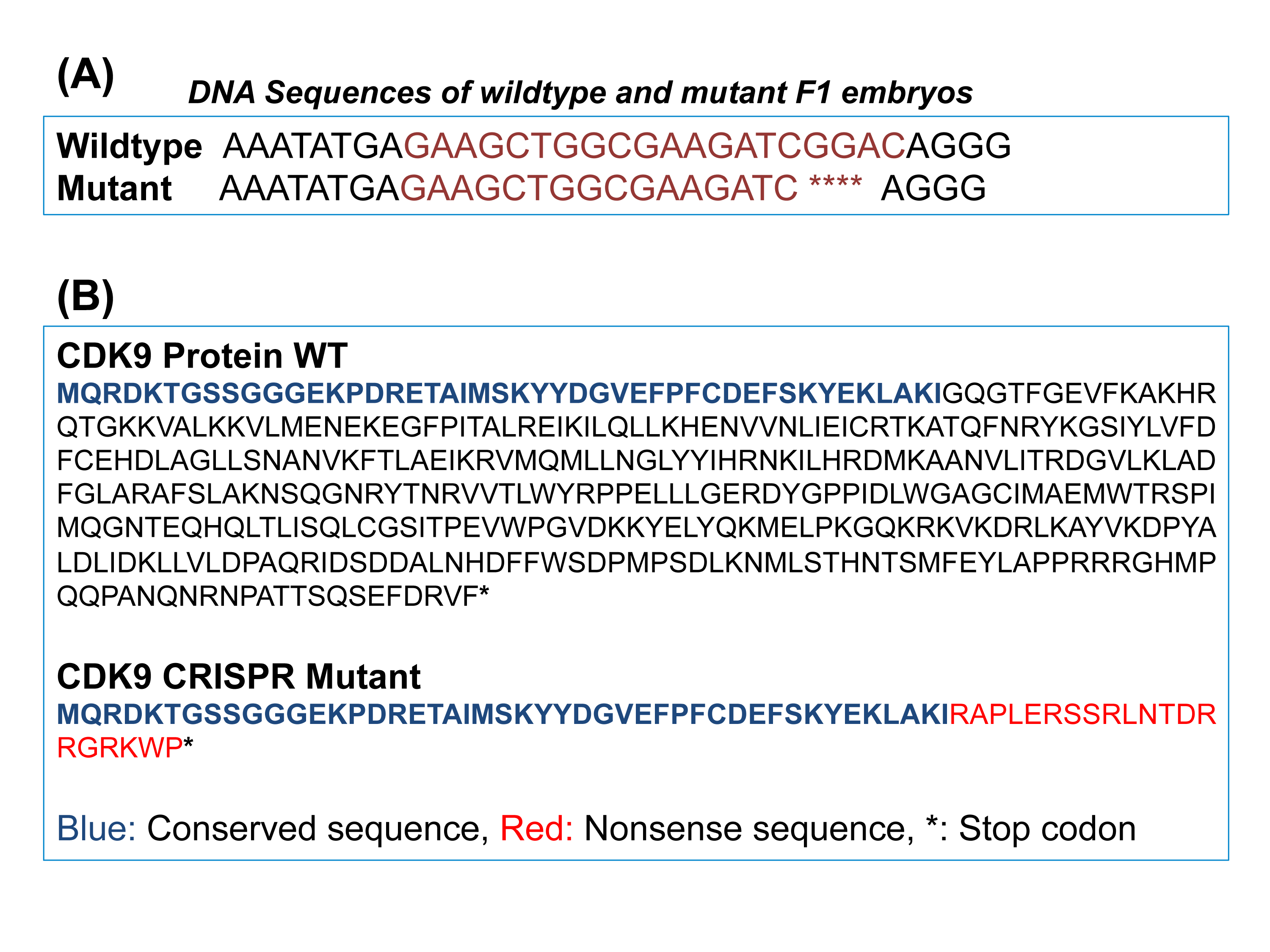
**

**Fig S3. Generating F1 heterozygote CDK9 knockout zebrafish using CRISPR/cas9.**

Fin clips from F1 fish (from a Tg[mpx:EGFP]^i114^ x CRISPR-injected Tg[mpx:EGFP]^i114^) were digested and sent for DNA sequencing; the sequence of the CDK9 exon 2 region is shown with the CRISPR target sequence in red (A). The protein sequence was determined from the DNA sequence, showing the conserved region between mutant and wild type (WT), and the early stop codon and nonsense sequence in the mutant (B).


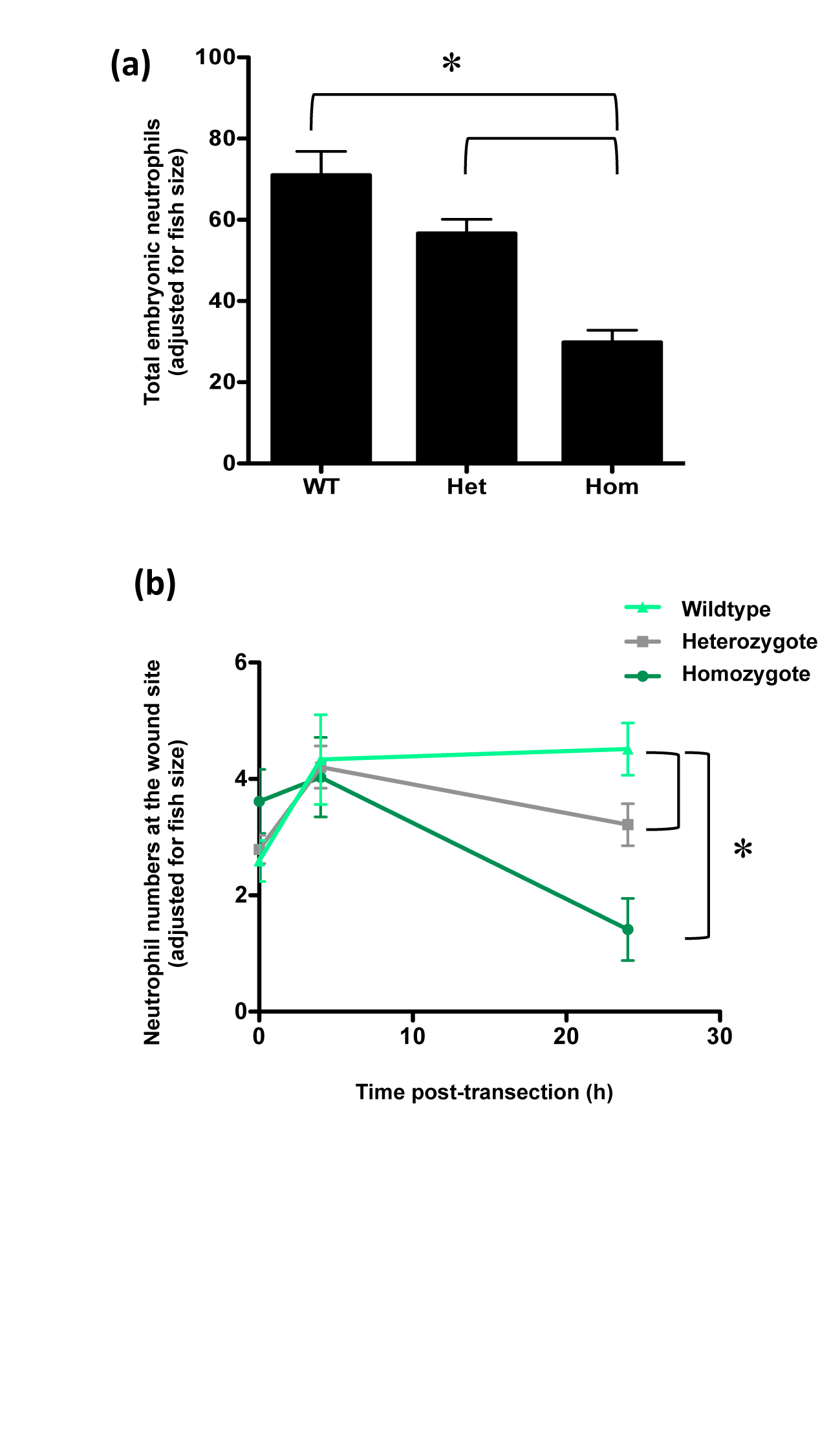


**Fig. S4. Adjusting neutrophil numbers for fish size in CRISPR/cas9 CDK9 knockout fish.**

(a) The body of the fish was measured from scale and the number of neutrophils in the whole fish per mm of fish was calculated in homozygote/heterozygote knockout and wild type fish. (b) Similarly, the numbers of neutrophils recruited to the wound in homozygote/heterozygote knockout and wild type fish were calculated with respect to fish size.
